# Research on SARS-COV-2 pandemic: a narrative review focused on the Italian contribution

**DOI:** 10.1186/s44158-021-00017-4

**Published:** 2021-11-17

**Authors:** Alessandro De Cassai, Federico Longhini, Stefano Romagnoli, Fabio Cavaliere, Antonio Caroleo, Lorenzo Foti, Elisa Furlani, Sara Gianoli, Francesco Monteleone, Giuseppe Saraco, Gianluca Villa, Giorgio Conti, Paolo Navalesi

**Affiliations:** 1 Anesthesia and Intensive Care Unit, University Hospital of Padua, Via Giustiniani 1, 35127 Padua, Italy; 2 Anesthesia and Intensive Care, “Mater Domini” University Hospital, Department of Medical and Surgical Sciences, “Magna Graecia” University, Catanzaro, Italy; 3 Department of Health Science, Section of Anaesthesia and Critical Care, University of Florence, Florence, Italy; 4 Department of Anaesthesia and Critical Care, Azienda Ospedaliero-Universitaria Careggi, Florence, Italy; 5 Department of Medicine-DIMED, University of Padua, Padua, Italy; 6 Department of Anesthesia and Intensive Care, Fondazione Policlinico A. Gemelli IRCCS, Rome, Italy

**Keywords:** COVID-19, SARS-COV 2, Italy, Review

## Abstract

**Background:**

Since late 2019, a severe acute respiratory syndrome, caused by the severe acute respiratory syndrome coronavirus-2 (SARS-CoV-2), has spread with overwhelming speed causing over 214 million confirmed infections and more than 4.5 million deaths worldwide. In this framework, Italy had the second highest number of SARS-CoV-2 infections worldwide, and the largest number of deaths. A global effort of both the scientific community and governments has been undertaken to stem the pandemic. The aim of this paper is to perform a narrative review of the Italian contribution to the scientific literature regarding intensive care management of patients suffering from COVID-19, being one of the first western countries to face an outbreak of SARS-CoV-2 infection.

**Main body:**

We performed a narrative review of the literature, dedicating particular attention and a dedicated paragraph to ventilatory support management, chest imaging findings, biomarkers, possible pharmacological interventions, bacterial superinfections, prognosis and non-clinical key aspects such as communication and interaction with relatives.

**Conclusions:**

Many colleagues, nurses and patients died leaving their families alone. To all of them, we send our thoughts and dedicate these pages.

## Introduction

Since late 2019, a severe acute respiratory syndrome, caused by the severe acute respiratory syndrome coronavirus-2 (SARS-CoV-2), has spread with overwhelming speed causing over 214 million confirmed infections and more than 4.5 million deaths worldwide [1].

SARS-CoV-2 was first identified in Wuhan, Hubei province, central China, and initial efforts were directed towards containing the diffusion of the virus; on January 13th, the first case reported outside of China was reported in Thailand. COVID-19 spread early in Italy too, with two Chinese tourists resulting positive to the virus on 31 January 2020 in Rome. On February 28th, WHO declared the global emergency risk level as “very high”. On March 12th, the global coronavirus disease-19 (COVID-19), the SARS-CoV-2-related infectious disease outbreak, was declared a pandemic.

**Fig. 1:**
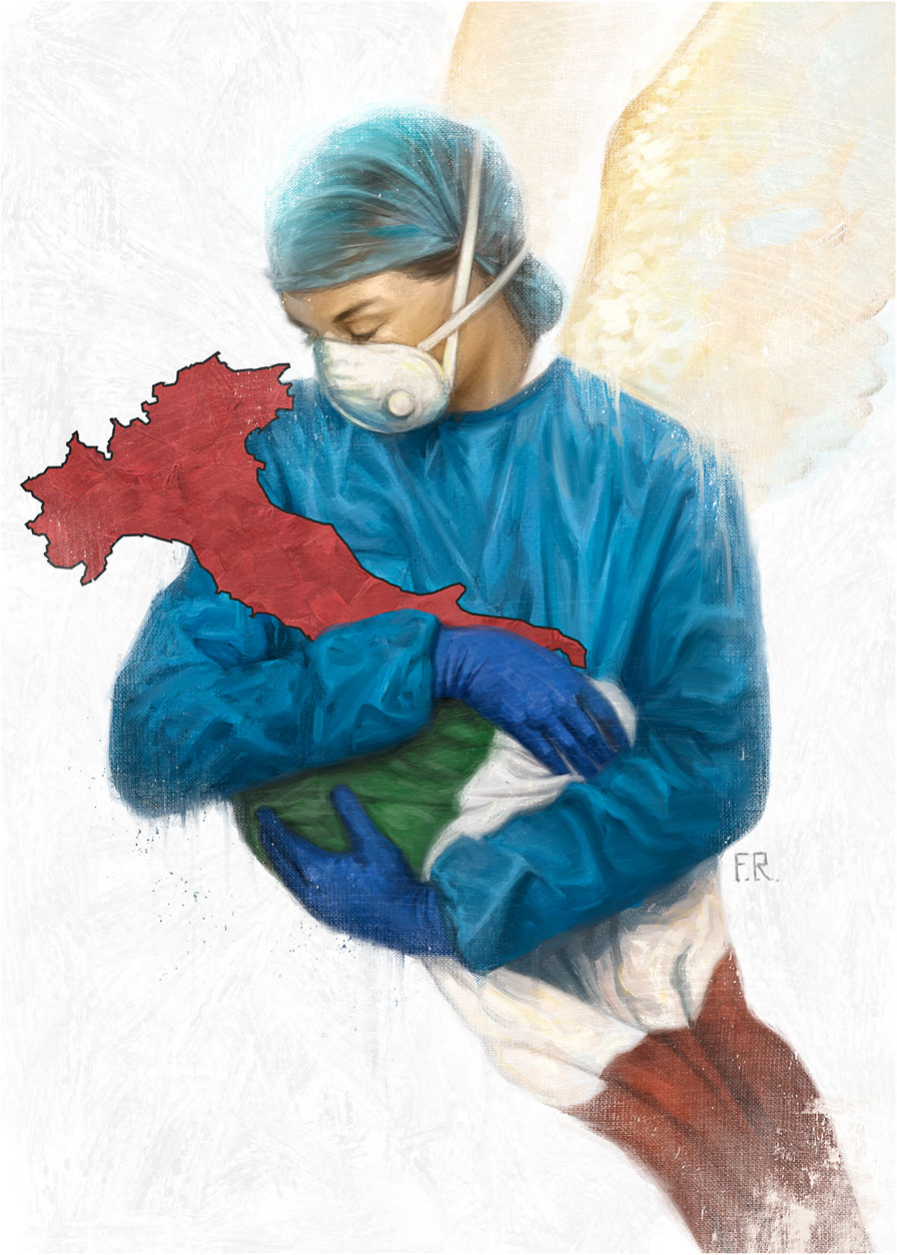
“Angels” by Franco Rivolli. Reproduced with permission from the author

A global effort of both the scientific community and governments has been undertaken to stem the pandemic. Several countries have swiftly adopted prevention and control measures to reduce the spread of COVID-19 transmission (e.g. physical distancing, staying at home recommendations and even lockdown) and the scientific community, in a race against the clock, tried to identify, propose and validate the most effective treatments for COVID-19. In this urge to retrieve an answer for the pandemic the early body of peer-reviewed COVID-19 literature was composed primarily of case series, observational studies and experts’ opinion, with randomized controlled trials lagging. The aim of this paper is to review the Italian contribution to the scientific literature regarding intensive care management of patients suffering from COVID-19, being one of the first western countries to face an outbreak of SARS-CoV-2 infection (Fig. 1).

## Main text

### Methods

A review protocol was written and agreed by all the authors before conducting this study.

The bibliographic search was conducted on June 23th, 2021, by one of the authors (ADC). In this review we included articles indexed by SCOPUS using the following string: “AFFILCOUNTRY (Italy) AND (TITLE-ABS-KEY(covid) OR TITLE-ABS-KEY (sars-cov-2)) AND (TITLE-ABS-KEY (ICU OR "intensive care")) AND PUBYEAR > 2018 AND NOT (TITLE-ABS-KEY ("case report")) AND (LIMIT-TO (DOCTYPE, "ar") OR LIMIT-TO (DOCTYPE, "re"))”.

Abstracts of the identified papers were screened to remove papers not concerning the SARS-CoV-2 topic; then the list of the remaining titles was reviewed with full-text retrieval for each paper. We included the papers regarding the SARS-CoV-2 pandemic, having an Italian corresponding author and investigating any aspect of intensive care unit (ICU) management, and we excluded all the papers related to pediatrics.

### Ventilatory support

SARS-CoV-2 infection is frequently complicated by hypoxemic acute respiratory failure (ARF), requiring oxygen supply and mechanical ventilation. Although considered a condition of acute respiratory distress syndrome (ARDS), COVID-19 is characterized by peculiar pathophysiological features with heterogeneous gas exchange and respiratory mechanics [2–4]. Severe hypoxemia might be related not only to loss of aeration and alveolar derecruitment but also to a disrupted ventilation/perfusion relationship [2]. Therefore, ventilator settings, including positive end-expiratory pressure (PEEP), should be personalized based on the patient’s conditions, and underlying vascular derangement [5].

The latest guidelines suggest oxygen supplement when SpO_2_ is < 92% to maintain a SpO_2_ below 96% [6]. In case of persisting hypoxemia, high-flow oxygen through nasal cannula (HFNC) is suggested over non-invasive ventilation (NIV), due to a reduced risk of intubation [6]. Of note, in critically ill patients with moderate-to-severe ARF, NIV through helmet has been shown to reduce the need for invasive mechanical ventilation (iMV), as compared to HFNC [7]. Therefore, an early trial with helmet NIV could be reasonable, though the evidence is weak. NIV application is also feasible outside the ICU to cope with the massive demand of ventilatory assistance [8]. However, close monitoring of respiratory function remains fundamental to not delay intubation, whenever needed [6]. In fact, it is known that in patients with hypoxemic ARF, intubation delay increases mortality. In patients receiving HFNC, the ROX index (pulse oximetry/fraction of inspired oxygen/respiratory rate) may help the clinician to early detect treatment failure, even outside the ICU [7]. After 12 h of HFNC, a ROX-index < 5.99 is associated with an increased risk of iMV in COVID-19 patients [7].

When iMV is instituted, it is recommended a protective ventilation strategy, consisting of a low tidal volume (4–8 mL/kg of predicted body weight), a plateau pressures < 30 cm H_2_O and a driving pressure < 15 cmH_2_O [6]. As mentioned above, PEEP selection should be individualized based on the lung conditions and respiratory mechanics [2, 5]. Computed tomography (CT) scan findings, together with respiratory mechanics, may also help the clinician to set the best PEEP, avoiding too high values which could be harmful [9, 10].

Despite the application of a protective ventilation strategy, air leak from the alveoli into the pleural cavity (pneumothorax) or mediastinum (pneumomediastinum) occurs frequently, and it is associated with increased mortality. Among reasons for pneumothorax and pneumomediastinum, the high inspiratory effort of COVID-19 patients plays a major role. The so-called patient self-inflicted lung injury produces high tidal volumes and high transpulmonary pressure, causing subtle damages which will become evident during NIV or iMV [4, 11, 12].

In case of persistent hypoxemia despite the optimization of ventilator settings, recruitment manoeuvres are suggested. Recruitment manoeuvres aim to open atelectatic alveoli and to improve oxygenation. However, recruitment manoeuvres expose the risk of barotrauma and transient hypotension [6]. Recruitment manoeuvres should not be performed using a staircase (incremental PEEP) strategy, and they are indicated only in patients with low lung compliance [2–5, 9, 10].

In mechanically ventilated patients affected by moderate-to-severe COVID-19 ARDS, prone positioning is also suggested for a 12- to 16-h session [6]. By redistributing pulmonary blood flow and reducing atelectasis (identifiable as loss of aerated areas on chest CT), Prone positioning improves oxygenation, reduces mortality, though an increased risk for pressure sores is present [9, 10]. Sustained oxygenation improvement after the first prone positioning session is independently associated with improved survival and it reduces the duration of iMV [13, 14]. It has been also proposed to prolong prone positioning sessions up to 36 h, since this is feasible and safe, it provides organizational advantages by reducing the number of prone positioning cycles for a single patient, and oxygenation improvement seems to be higher [15].

PP has been also proposed as an early strategy to improve gas exchange and reduce the risk for iMV in awake patients during HFNC and NIV [16–21]. However, data are conflicting: although no difference in the intubation rate has been shown between responders and non-responders [20], a retrospective multicenter cohort study suggests that PP can reduce the need for iMV when applied to patients receiving HFNC, but not in those with NIV support [18]. In addition, in patients undergoing NIV, awake PP improves gas exchange at the expenses of an increased diaphragm activation and worsening of the comfort [20]. NIV can also be successfully used to facilitate weaning from iMV. As compared to conventional weaning strategies, an early extubation with NIV application reduces the time spent under iMV, extubation failure rate and number of reintubation. No differences were found between strategies with respect to rate of tracheostomy, ICU mortality and length of stay [22]

In moderate-to-severe COVID-19 ARDS undergoing iMV, the use of neuromuscular blocking agents is also suggested to facilitate the protective ventilation strategy, preferring whenever possible intermittent dosing over continuous infusion [6].

Inhaled nitric oxide can be also considered as a rescue strategy in mechanically ventilated patients with severe ARDS; however, if no rapid improvement in oxygenation is observed, the treatment should be tapered off [6]. In patients with elevated lung compliance, inhaled nitric oxide may potentially improve oxygenation by acting on lung perfusion but no data are available from this population [9]. Its usefulness is more appropriate in case of pulmonary hypertension [10].

### Chest imaging

Chest imaging plays a crucial role in the diagnosis and in the follow-up/management of patients with COVID-19 pneumonia.

Although the gold standard is the CT scan, the need to transfer unstable COVID-19 patients to the radiology department, the risk of viral spread and the high costs limit the applicability of this technique. In this scenario, bedside imaging techniques, such as portable chest X-ray (CXR), and lung ultrasound (LUS) could be alternatives in varying conditions and for different aims.

#### Chest X-ray

The American College of Radiology pointed out that the use of CT scan may disrupt radiological service availability due to the CT room decontamination [23], Therefore, diagnostic algorithms have been proposed for CXR, as a first-line triage tool, in patients with clinical suspicion and waiting for results of nasopharyngeal swabs [23, 24]. CXR is also suggested in the routine follow-up to obviate the need for CT [24].

However, in COVID-19 patients, findings may be nihil in the first days of disease. Ten to twelve days after the onset of symptoms, CXR can detect bilateral ground glass or patchy opacities, uncommonly associated with pleural fluid [23, 25]. Indeed, sensitivity is quite heterogeneous, ranging from 33 to 84%, according to the timing and disease severity [25]**.** The application of artificial intelligence (AI) to CXR at admission allows to compute radiological scores predicting critical disease and mortality. In particular, a Qure AI score ≥ 30 or a Radiographic Assessment of Lung Edema score ≥ 12 on the CXR at presentation are independent and comparable predictors of adverse outcomes [26]. The semi-quantitative CXR score (ranging from 0 to 18) has been also proposed in a preliminary study to describe the disease severity [27].

Advantages of CXR are the low cost, the bedside availability with a dedicated portable CXR machine and the reduction of the risk of virus spread related to patient’s transport, while the major limitations are the low sensibility and the low correlation between radiological findings and disease progression [24].

#### CT scan

The role of CT for the diagnosis of COVID-19 interstitial pneumonia is well established. CT scan is more sensitive and specific than CXR; it can identify interstitial involvement of lung parenchyma, even in the early stage of the disease [25]. Typical features on chest CT are ground-glass opacities (with a prevalence between 46 to 100%) and consolidation (in the later stages of the disease), frequently associated with other ancillary findings [25, 28, 29]. Furthermore, CT scan can highlight the involvement of the lung vascular system, as well as common vascular events other than pulmonary embolism [30]. CT scan is indicated for COVID-19 patients presenting with moderate to severe clinical features and to identify worsening of lung disease [29]. Noteworthy, the extension of lung involvement, as assessed by the quantitative Pulmonary Involvement score, can predict the disease worsening, ICU admission and patients’ mortality [31].

#### Lung ultrasound

LUS is a widely used bedside imaging technique to explore pleura and the entire lung parenchyma, following a 12-zone scanning protocol [24].

Ground glass opacities and reticular pattern on CT scan are represented by bilateral, irregularly distributed B-lines with spared areas (B-1 pattern) and coalescent B-lines (B-2 pattern) at LUS. Along disease progression, subpleural consolidations increase, as identified by anechoic hemispheric areas close to the pleural line with a hyperechogenic base. Extensive consolidations appear at LUS as consolidation with hepatization of lung tissue and air bronchogram (C pattern) [23, 25, 32–34]. LUS score (as computed by the sum of scores for each parenchyma area according to the findings) is increased at ICU admission, but remains elevated at ICU discharge [32]. In addition, LUS score at admission and 72 h after hospital admission are reliable prognostic tools for worsening of respiratory failure and need for continuous positive airway pressure (the former) [32, 35], need for ICU admission and risk of death (the latter) [35]. Lung and cardiac combined ultrasounds are also useful to monitor the disease progression and to identify parameters (LUS score and right ventricle/left ventricle ratio) to stratify the risk of death [36]. LUS has been also suggested in integrated extubation protocols, to monitor lung aeration before and after extubation, and to predict extubation failure [37].

LUS has the advantage of bedside serial monitoring, to reduce the risk of cross-infection and virus spread related to patients’ transport [24] and the number of CXRs and CT scans [38]. Disadvantages are that LUS is operator-dependent and time-consuming, needs operator experience to generate high-quality and reproducible images and does not detect abnormalities affecting the central regions surrounded by aerated lung [23, 24].

### Biomarkers

Biomarkers can be intended as almost any measurement reflecting an interaction between a biological system and a potential hazard, which may be chemical, physical, or biological. The measured response may be functional and physiological, biochemical at the cellular level, or a molecular interaction as defined by WHO. In a new unknown disease such as COVID-19 the study of biomarkers has shown to be extremely useful for understanding the physiopathology of the infection, identifying the prognostic factors and addressing the therapeutic strategies.

#### Vitamin D

25-Hydroxyvitamin D (25OHD) has been, from the early moments, a strongly debated protagonist among the variety of biomarkers associated with SARS-CoV-2 infection. Its known modulatory role in both innate and adaptive immunity, including the downregulation of inflammatory cytokines such as interleukin-6 (IL-6), together with the observation of its protective capability for infections of the respiratory tract, have led to several studies regarding this molecule. Even though a univocal conclusion has not been reached, it has been shown that circulating levels of 25OHD were lower in severely symptomatic COVID-19 patients than in mildly symptomatic subjects [39]. Such studies made it possible to identify 25OHD as a plausible independent prognostic factor and open the way to further studies on the effects of supplementation with vitamin D in deficient individuals.

#### Coagulation

The severe phenotype of SARS-CoV-2 infection is frequently associated with elevated D-dimer [40] low platelet count [41], together with elevated von Willebrand factor and higher agglutination rates induced by ristocetin [42]. These features are strongly suggestive of systemic clotting activation and enhanced agglutination, with secondary fibrinolysis and thrombocytes consumption. These findings have demonstrated to have important consequences both from the prognostic point of view, being elevated D-dimer and low platelets associated with a worse prognosis, and the therapeutic one, since prophylaxis with low molecular weight heparin has resulted in better outcomes and higher survival rates [40, 41].

#### Inflammation

IL-6 is a pleiotropic, proinflammatory, multifunctional cytokine released by different types of cells, including macrophages, adipocytes and blood cells. Produced during inflammatory processes, IL-6 is also fundamental for production of another important biomarker such as C-reactive protein, so the two molecules are strongly linked.

IL-6 can be considered an early biomarker in the prediction of clinical deterioration of patients with moderate-to-severe COVID-19, especially when associated with other biochemical and physiological parameters such as SpO_2_/FiO_2_ ratio and C-reactive protein itself [43]. Moreover, playing such a pivotal role in the pathogenesis of moderate-to-severe SARS-CoV-2 infection, IL-6 can also represent an important target for therapeutic strategies involving monoclonal antibodies (i.e. Tocilizumab) [44–46].

C-reactive protein, as mentioned before, has also shown increased levels during SARS-CoV-2 infection and higher values have appeared to be strongly associated with a higher risk of ICU transfer [47, 48].

Moreover, procalcitonin has also emerged to be an adverse outcome index, especially in ICU, when its elevated values can be also correlated to bacterial superinfection [40].

#### White blood cells

Leucocytes show a peculiar pattern in moderate-to-severe infection from SARS-CoV-2. This pattern is characterized by leukocytosis accompanied by a major increase in neutrophils count [49] and a relevant lymphopenia [44, 50].

Neutrophilia can be explained by the severe Systemic Inflammatory Response Syndrome and the cytokines storm that accompany ICU patients, but also by potential superinfections [49, 51]. On the other hand, the decrease of lymphocytes count can find an explanation in the numerous data suggesting tissue sequestration (especially in lungs, lymphoid tissues and GI tract) and destruction in peripheral blood, due to the prevalence of a senescent phenotype of B cells, T cells and natural killer-NK [44, 51].

An important implication of this specific pattern is the possibility to use a particular parameter, the neutrophil-to-lymphocytes ratio, as an independent predictor of severe COVID-19 cases that require ICU admission [51].

#### Organ damage

Firstly, liver enzymes have proved to be frequently elevated in severe SARS-CoV-2 infection. Whether this is due to direct or hypoxic damage is not clear, but elevation of liver enzymes has shown association with poorer outcome [52]. Heart damage has also appeared to be a common laboratory finding: approximately 50% of patients have elevated cardiac biomarkers at admission and elevation of both highly sensitive troponin-I (hs-TnI) e brain natriuretic peptide (BNP) has appeared to be a strong independent predictor of mortality [53]. Ultimately, lactate arterial-venous gradient is a parameter that has emerged to be a valid biomarker of lung damage, especially in mechanically ventilated patients. An augmented gradient, in fact, has appeared to be an expression of lung inflammation and can be a useful monitoring tool [49].

### Pharmacological interventions

Despite an increase in knowledge over SARS-COV-2 and global efforts to identify interventions for the prevention and treatment of COVID-19, there is evidence for effective treatment protocols. Interesting possibilities are emerging thanks to the use of drugs that were originally developed for other clinical indications, whose pharmacological profiles suggest an impact on some of the multiple pathophysiological aspects of COVID-19 illness.

#### Low-molecular-weight heparin

Patients with SARS-COV-2 infection have a high risk to develop venous thrombotic events [54] and since the beginning of the pandemic, the WHO has recommended antithrombotic prophylaxis with low molecular weight heparin to reduce the venous thromboembolism risk.

A prospective study including 264 patients who did not require ventilation has shown that prophylaxis significantly and independently reduced mortality [55].

High mortality in ICUs, data from autopsies of ICU patients indicated pulmonary embolism as a frequent fatal event. The observation of several risk factors for deep vein thrombosis in patients hospitalized with COVID-19 prompted some ICUs to increase the prophylactic dose of enoxaparin from 40 mg daily up to 1 mg/kg twice daily. Results show that patients receiving high doses of enoxaparin have reduced mortality, clinical deterioration and venous thromboembolism by approximately 50–60% compared to patients on standard doses [56]. The optimal prophylactic enoxaparin dose is still debated.

It is still not clear which are the best tests to monitor and guide anticoagulation in acutely ill patients, Anti-Xa seems a more reliable method to monitor heparin treatment in acute patients [57].

#### Corticosteroid

The efficacy and safety of corticosteroids in patients with SARS-COV-2 infection are still debated. An observational prospective study on patients admitted for COVID-19 who did not receive iMV shows that treatment with corticosteroids is not associated with a reduction of in-hospital mortality; although within the subgroup of COVID-19 patients treated with NIV, corticosteroid therapy is associated with a lower odds ratio for ICU admission [58, 59]. According to this multicenter observational study, early administration of prolonged, low dose methylprednisolone treatment in patients with severe COVID-19 pneumonia was associated with a significantly lower risk of death and decreased ventilator dependence. Methylprednisolone treatment was demonstrated to be safe and also allowed for a significant decrease in mortality and an immediate improvement in systemic inflammation and oxygenation markers, as well as a reduction of iMV length [60].

A Meta-Analysis of randomized controlled trials shows that corticosteroids may be considered in critically ill patients with COVID-19 but must be discouraged in patients not requiring oxygen therapy: it appears that spontaneously breathing patients treated with corticosteroids had less requirement for iMV, but higher mortality rate than patients who did not receive corticosteroids [61].

#### Tocilizumab

Tocilizumab has become one of the therapeutic options for the management of cytokine release syndrome, a condition that seems to be present also in patients hospitalized with respiratory failure due to COVID-19, therefore it was hypothesized that tocilizumab could be clinically effective for this population. So far, the experience with tocilizumab in COVID-19 patients is limited and studies show different results due to a lack of a standardized therapeutic scheme, short post-treatment follow-up and the absence of a comparison group. This retrospective cohort study did not show a clear improvement in patients receiving tocilizumab compared to standard management [62] although other studies including severe COVID-19 patients admitted to ICU seem to show an improvement in their clinical status and the need for iMV [63, 64].

#### Remdesivir

Remdesivir treatment seems to have a beneficial effect on SARS-CoV-2 pneumonia, especially in the case of non-critically ill patients [65]. Remdesivir does not appear to be associated with a significant reduction of mortality in mechanically ventilated patients, but it is consistently associated with a shorter duration of iMV and higher probability of hospital discharge, independently of other risk factors [66]. Contrarily, in a different study Remdesivir appears to be associated with a significant beneficial effect on the survival of patients with COVID-19 treated with iMV [67]. Ongoing randomized controlled trials are needed to clarify its efficacy, safety, whom to treat and when.

### Bacterial superinfections (or secondary infections)

Bacterial superinfections seem to play a crucial role in COVID-19 patients admitted to the ICU, acting as a key determinant of important outcomes such as ICU and hospital length of stay, duration of iMV and mortality. Current literature shows that bacterial superinfections affect a significant number of patients, although there is a wide variation in the reported incidence of this complication [68, 69]. In the light of the above findings, data on the incidence of superinfections should be interpreted and analysed with caution since most COVID-19-related studies have not included superinfections among study endpoints. Italian physicians have efficiently contributed to spread knowledge on this topic, with data reports and publications focusing on drug-resistant bacteria. In a recent review on secondary infections, Fattorini and collaborators, underlined that secondary infections significantly decreased survival of COVID-19 patients, especially if they had been admitted to the ICU [70]. De Santis and colleagues conducted a prospective observational study of critically ill patients with COVID-19 admitted to eight Italian ICUs [69]. In line with previous evidence, the authors observed that, of the 248 patients recruited, 90 (36.3%) developed at least one episode of secondary infection. Increasing ICU length of stay was associated with a higher occurrence of infectious complications, with ventilator-associated pneumonia (VAP) being the most frequent. In patients with bacteremia and VAP, a balanced mix of Gram-negative and Gram-positive organisms was found, although polymicrobial and urinary tract infections were more frequently caused by Gram-negative microorganisms. At least one course of antibiotic therapy was given to 161 (64.9%) patients. Logically, patients developing bacteremia had a higher risk of ICU and hospital mortality (45.9% vs. 31.6% and 56.8% vs. 40.3%, respectively). Based on these findings, the authors concluded that regular microbiological surveillance and strict infection control measures should be implemented for the management of COVID-19 patients. A multicenter, before-and-after, cross-sectional study compared the rates of colonization and infection with carbapenemase-producing *Enterobacteriaceae* and/or carbapenem-resistant *AcinetobacterBaumannii* in two different study periods (Jan–Apr 2019 vs. Jan–Apr 2020) [71]. Interestingly, compared to period 1, during period 2 the incidence rate ratios of colonization and infection with carbapenem-resistant *Acinetobacter Baumannii* increased 7.5- and 5.5-fold, respectively. Differently, no significant changes were found in carbapenemase-producing *Enterobacteriaceae* colonization and infection during the two study periods. Concerning the topic of multi-drug resistant (MDR) bacteria secondary infection, Pasero and collaborators performed a review of the literature to investigate the incidence of this important co-infections/superinfections [72]. According to the authors, most of the studies (mainly retrospective and single-centred) reported low rates of co-infections but a significant incidence of hospital-acquired infections developing about 10–15 days after ICU admission. In fact, most bacterial infections described in the COVID-19 population emerged during the ICU and hospital stay (secondary bacterial infections), with an estimated incidence of 14.3% [70]. The reported incidence of MDR bacterial secondary infections was high (ranging between 32% to 50%). Pasero and colleagues suggested that prolonged ICU length of stay and extensive use of broad-spectrum antimicrobial drugs might have contributed to the selection of pathogens. Similarly, MDR infections were associated with a longer ICU stay. IMV was identified as a risk factor independently associated with MDR secondary infections. Ultimately, the authors recommended judicious prescription and management of antimicrobials according to a stewardship program. Relative to investigation strategies, Foschi and collaborators assessed respiratory bacterial co-infections in lower respiratory tract samples by comparing the conventional culture approach to an innovative molecular diagnostic technology based on multiplex PCR panel (FilmArray Pneumonia Plus panel) [73]. More than 30% of samples were culture positive for pathogens, including *Pseudomonas aeruginosa*, *Klebsiella pneumoniae* and *Staphylococcus aureus*. Film-Array showed an overall sensitivity and specificity of 89.6% and 98.3%, respectively, with a negative predictive value of 99.7%. According to the authors, the molecular test significantly reduced the turn-around-time and increased the rates of microbial detection but missed a list of pathogens not included in the molecular panel, especially *Stenotrophomonas maltophilia*. A retrospective study investigated possible predictive factors for bacterial VAP in a cohort of 39 COVID-19 patients who required iMV [74]. Fifty-four percent of the patients were diagnosed with bacterial VAP. Multivariate logistic regression for prediction of VAP showed significant differences in duration of ICU hospitalization and in minimal lung compliance. Additionally, 71% of the isolated germs were MDR and bacteremia was reported in 38%. Multivariate analyses for prediction of lethality found significant differences in sequential organ failure assessment (SOFA) score. Cataldo and collaborators performed a retrospective cohort study including adult COVID-19 patients hospitalized in ICU at the National Institute for Infectious Diseases, Rome, Italy, to assess the incidence of bacterial and fungal bloodstream infections (BSIs) in COVID-19 patients [75]. Data collected in this study evidenced an exaggerated risk of acquiring bacterial and fungal BSIs among critically ill COVID-19 patients admitted to the ICU. In the pre-COVID-19 period, the prevalence of BSIs in patients staying at the same ICU was 3.8 times lower than the prevalence observed in the study. Antinori and colleagues published a review on studies reporting bacterial and fungal co-infections in patients with COVID-19 [76]. Most studies were retrospective and produced poor quality data biased with short follow-up and selection of patients. Septic shock was reported in 4% to 33.1% of patients and 71–100% of patients received antimicrobial therapy. Interestingly, the authors observed that, as previously reported in the influenza pandemic, invasive pulmonary aspergillosis seems to be an increasingly observed complication in critically ill patients with SARS-CoV-2 infection. In conclusion, hospitals managing surges of patients with COVID-19 eventually are vulnerable to outbreaks of MDR organism infections. Developing dedicated infection prevention and control best practices, to the largest extent possible, is mandatory and there is a need for careful infection control activities targeted against the spread of antimicrobial resistance.

### Prognosis

The disease severity and mortality in patients with COVID-19 has been extensively studied and several variables have been investigated as independent risk-factors for worsened outcomes.

First of all, patients with cardiometabolic comorbidities are at increased risk for worsened outcomes [77]. Cerebrovascular and cardiovascular co-morbidities, chronic obstructive pulmonary disease, diabetes, hypertension, smoking and male sex are associated with severe disease and mortality [77, 78]. Newly diagnosed diabetes and admission hyperglycemia are powerful predictors of COVID-19 severity due to rapid respiratory deterioration [79]. Hyperglycemia (>180 mg/dl) is more common in non-survivors showing also a significantly higher glucose variability in the first 48 h after ICU admission [80]. Obesity is another independent risk factor for respiratory failure, hospital admission, more severe progression of the disease requiring ICU admission and death [81, 82]. A body mass index higher than 30 kg/m^2^ identifies a population of patients at high risk for severe illness, whereas a value higher than 35 kg/m^2^ dramatically increases the risk of death [81]. Furthermore, the presence of visceral fat is significantly higher in patients requiring ICU admission, it is a marker of worse clinical outcomes in patients with COVID-19, as well as it prolongs the hospital and ICU lengths of stay and increases the risk of death [83, 84].

In the context of critically ill COVID-19 patients, lymphocytopenia, thrombocytopenia, ferritin, liver enzyme elevation, high D-dimer and lengthening of prothrombin time are strictly correlated with higher ICU and/or hospital mortality [40, 52, 85, 86].

The presence of increased procalcitonin, increased D-Dimer and thrombocytopenia also predict the occurrence of severe infections [41]. In addition, low serum prealbumin concentrations are significantly associated with COVID-19 severity and mortality. This combined marker of malnutrition and inflammation might assist with early risk stratification and management in this group of patients [87].

Serum sodium at admission may be considered as an early prognostic marker of disease severity in hospitalized COVID-19 patients. Patients with hyponatremia have a higher need for non-invasive ventilation and ICU admission than those with normonatremia or hypernatremia. Hyponatremia is also an independent predictor of in-hospital mortality, and each mEq/L of serum sodium reduction is associated with a 14.4% increased risk of death [88].

25OHD and IL-6 levels are also associated with poorer outcomes. In particular, 25OHD and IL-6 levels are respectively lower and higher in severely symptomatic COVID-19 patients admitted to ICU, as compared to not critically ill patients, and in non-survivors as compared to survivors [38, 89]. Hypovitaminosis D is also associated with susceptibility to respiratory infections [89].

In COVID-19 ICU-patients, mid-regional pro-adrenomedullin (MR-proADM) seems to have constantly higher values in non-survivors and predict mortality more precisely than other biomarkers. Repeated MR-proADM measurement may support rapid and effective decision-making [86, 90].

A COVID-19 in-hospital mortality risk score is a clinical risk score proposed to predict the in-hospital mortality, based on a set of variables available soon after the hospitalization. Included predictors are age, number of chronic diseases, respiratory rate, PaO_2_/FiO_2_, serum creatinine and platelet count. It is highly accurate in stratifying patients at low, intermediate and high risk of in-hospital death [91].

Finally, in patients with septic shock, low levels of circulating immunoglobulins are common, and their kinetics appear to be related to clinical outcome. Regarding adjunctive therapy with polyclonal immunoglobulins, several studies have revealed that immunoglobulin titles are commonly low at the onset of sepsis and that during the sepsis course. Persistent low levels of immunoglobulins are closely related to increased mortality [92].

### Communication and relatives

The urgent ICU admission of patients with COVID-19 is undoubtedly a stressful event with significant psychological repercussions on patients’ family members. Despite the efforts made during the COVID-19 pandemic to improve communication and personal interactions among patients, relatives and healthcare workers, an incredibly high prevalence of burnout, post-traumatic, major depressive, or generalized anxiety disorders and pathological or complicated grief has been observed [93].

Several authors have critically explored how to communicate with and involve families living in complete isolation in the last years. Clinical communication in this context should provide understandable information about the disease and treatment options, obtain information on expectations and choices, show collaboration, allow relatives to express their emotions, prevent misunderstandings and conflicts with the care team. Indeed, specific technical and non-technical skills in communication are required to reach these targets. Critical importance has been recognized for the relational aptitude and the preparation of the communication, confidentiality, truth, coherence, gradualness and grief. Information on patients’ clinical conditions should reach relatives at least once a day, at the same time, from a quiet and suitable place, preferably from the same doctor who knows the patient well. As the healthcare workers’ mental and emotional well-being plays a key role in this process, psychological assistance should always be considered for both operators and relatives [94]. Of note, particularly in this pandemic course, the assistance to relatives should be continued during their grief [95], mainly for the restrictions in seeing, visiting, or performing funerals to the loved one.

Peculiar characteristics have also been recognized for the modalities of communication. The introduction of physical isolation measures during the SARS-CoV-2 pandemic has profoundly changed communication with relatives, almost entirely based on telephone calls. When their clinical conditions allow the patients to interact with relatives, video calls with dedicated tablets and smartphones have been applied to reduce social distances. The same technology has been applied to support the patient with external specialist consultations as psychological therapies [96]. Finally, enhanced strategies for telecommunication in the ICU have allowed the health workers to establish a more direct relationship with the families, provide them more precise information on patients’ clinical condition and prognosis, encourage discussion on patients’ preferences or expectations at the end-of-life and ultimately promote shared decision-making.

#### Organization

The unexpected and sudden increase in iMV and multiple organ support requirements in COVID-19 patients [97] has forced the hospitals to reallocate the available resources, substantially modify their infrastructures and entirely reconsider their activities [98–100]. For example, except for transplantations, oncological and un-deferrable surgeries, the elective surgical procedures were almost cancelled in every Italian region [101]. Healthcare staff involved in the perioperative medicine was thus reassigned to critical care, and more beds in the postoperative ICU became available. Furthermore, the same operating theatres and anaesthesia recovery units have been reallocated as intensive care or high-dependency units in many cases [102].

The increase in the ICU beds capacity was undoubtedly the priority during the COVID-19 outbreak. Accordingly, the extensive hiring campaign performed by several hospitals (made possible by the increase in funds dispatched by the national health system) has been addressed towards healthcare workers primarily involved in emergency and critical care.

A careful separation between COVID and non-COVID pathways was maintained to minimize the risk of in-hospital SARS-CoV2 infection. In this context, creating dedicated high-dependency units and triage areas where patients with respiratory symptoms could be treated waiting for the SARS-CoV2 diagnostic tests before being assigned to a specific department was crucial to relieve ICU from the risk of overload [103, 104]. Interestingly, many hospitals had the opportunity to use buildings dedicated exclusively to COVID patients, especially during the second wave of COVID-19 infection (October 2020).

### Conclusions

By the end of March 2020, Italy had the second highest number of COVID-19 infections worldwide, and the largest number of deaths. After the identification of the first severe case of COVID-19 on February 20, 2020—a young man with no history of possible exposure abroad, diagnosed with COVID-19 in Codogno, Lombardy—the outbreak rampaged through various areas of northern Italy. Within 2 weeks, an exponential increase in new cases of COVID-19, including many critically ill patients, was reported in the surrounding areas, and new clusters were identified in the nearby regions of Piedmont and Veneto. Since then, COVID-19 infection has spread throughout the country with a somewhat lower impact from north to south. The Italian anaesthesiologists/intensivists, nurses and many other professionals and institutions (e.g. engineers, companies) faced something never seen before. This review of the literature is dedicated to all of them that with invaluable courage, dedication, efforts and sacrifice have dedicated their professional life to thousands of patients. Many colleagues, nurses and patients died leaving their families alone. To all of them, we send our thoughts and dedicate these pages.

## Data Availability

Data sharing is not applicable to this article as no datasets were generated or analysed during the current study.

## Abbreviations

25OHD: 25-Hydroxyvitamin D
ARDS: Acute respiratory distress syndrome
ARF: Acute respiratory failure
CXR: Chest X-ray
CT: Computed tomography
COVID-19: Coronavirus disease-19
HFNC: High-flow oxygen through nasal cannula
ICU: Intensive care unit
IL-6: Interleukin-6
iMV: Invasive mechanical ventilation
LUS: Lung ultrasound
MR-proADM: Mid-regional pro-adrenomedullin
MDR: Multi-drug resistant
NIV: Non-invasive ventilation
PEEP: Positive end-expiratory pressure
SARS-CoV-2: Severe acute respiratory syndrome coronavirus-2
VAP: Ventilator-associated pneumonia
